# ﻿Larval and adult morphology of *Photuriselliptica* Olivier (Coleoptera, Lampyridae) and a Halloweeny case of cave-dwelling firefly larva feeding on bat guano

**DOI:** 10.3897/zookeys.1203.120341

**Published:** 2024-05-28

**Authors:** Paula M. Souto, Simone P. Rosa, Robson de A. Zampaulo, Sara C. Rivera, Thais G. Pellegrini, Luiz F. L. da Silveira

**Affiliations:** 1 Centre for Functional Ecology, Department of Life Sciences, Associated Laboratory TERRA, University of Coimbra, Coimbra, Portugal University of Coimbra Coimbra Portugal; 2 Instituto de Recursos Naturais, Universidade Federal de Itajubá, Itajubá – MG, Brazil Universidade Federal de Itajubá Itajubá Brazil; 3 Observatório Espeleológico, Belo Horizonte, Minas Gerais, Brazil Observatório Espeleológico Belo Horizonte Brazil; 4 Western Carolina University, Biology Department, 1 University Drive, Cullowhee, NC 28723, USA Western Carolina University Cullowhee United States of America; 5 Laboratório de Ecologia Florestal, Departamento de Ciências Florestais, Universidade Federal de Lavras, Lavras, Brazil Universidade Federal de Lavras Lavras Brazil

**Keywords:** *Bicellonycha*, cave fauna, coprophagy, Photurinae, predatory fireflies

## Abstract

The predatory firefly *Photuriselliptica* is common throughout the Atlantic Forest and has been proposed as a biomonitor due to the species’ narrow niche and elevational range. However, the species is only known from adults, and a more effective monitoring of its populations hinges on the lack of knowledge on their immature stages. Recent sampling in ferruginous caves and inserted in other lithologies, on sites in the Atlantic Forest and Cerrado, have led to the capture of firefly larvae later reared to adults in the lab. Firefly larvae have been reported in South American caves before; however, they have only been identified to family due to the adult-biased taxonomy of Lampyridae. Here, we provide an updated diagnosis of *Photuriselliptica*, describe its immature stages for the first time, and update the distribution of the species. The larvae of *Photuriselliptica* were observed to interact with guano of several bat species, including that of vampire bats. These observations are consistent with the less specialized feeding preferences of photurine larvae, unlike most other firefly taxa, which specialize in gastropods and earthworms. It is yet unclear whether *P.elliptica* are cave specialists. However, since its occurrence outside caves remains unknown, protecting cave environments must be considered in conservation strategies for this important biomonitor species.

## ﻿Introduction

Fireflies (Coleoptera, Lampyridae) spend most of their lives as larvae, when they specialize on eating soft-bodied invertebrates such as gastropods and earthworms (Riley et al. 2021). For most species, except for the predatory ones (subfamily Photurinae), this is the only part of their life cycles responsible for obtaining and incorporating nutrients, since the adults usually do not eat (Faust 2017; Souto et al. 2019). Yet, firefly larvae tend to have highly diverse and specialized habitat preferences, including aquatic (freshwater, marine, or brackish water), semi-aquatic (in marshes, ponds, or bromeliads), and terrestrial (in leaf litter or soil) environments (reviewed by Riley et al. 2021). Given the importance of understanding and conserving firefly species, it is surprising that the immature stages of 94% of all firefly species remain completely unknown. Therefore, studies documenting the occurrence, behavior, morphology, and life cycle of larval forms are needed to fill out this Haeckelian gap (Faria et al. 2021).

The predatory fireflies in the genus *Photuris* Dejean, 1833 have been extensively studied for their complex adult behaviors (reviewed by Faust 2017; Lloyd 2018; Souto et al. 2019; Maquitico et al. 2022), remarkably including kleptoparasitism (Faust et al. 2012) and aggressive mimicry (“femme fatale”) (Lloyd 1965; Buschman 1974; Lloyd and Ballantyne 2003). This genus is divided in three subgenera, including Photuris (Photuris) commonly found throughout the New World from Canada to Argentina, with about 120 species (Olivier 1886; McDermott 1966; Souto et al. 2019; Heckscher 2021, 2023; Perez-Hernandez et al. 2022). However, their immature counterparts are comparatively neglected from a systematic standpoint despite their usually high local abundance, and studies on material reliably identified to species level are scarce (despite important work on ultrastructural morphology; e.g. Smith 1963; Strause et al. 1979). Aside from Rosa’s (2007) detailed work on the morphology and bionomy of *Photurisfemoralis* Curtis, 1839 (there misidentified as *Photurisfulvipes* (Blanchard, 1837)), comparative works of taxonomic relevance are lacking for this genus, and for subfamily at large (but see Costa et al. 1988, for descriptions of the immature stages of an undetermined species of *Bicellonycha* Motschulsky, 1853). Studies on undetermined (i.e. unidentified to species) *Photuris* larvae (e.g. Oertel et al. 1975; Domagala and Ghiradella 1984; Murphy and Moiseff 2019) highlight the challenge of identifying larvae and the need for comparative work on reliably identified species to foster further studies on this group.

The predatory firefly *Photuriselliptica* Olivier, 1886 has been identified as an ideal flagship species to monitor environmental changes in the Atlantic Rainforest, given their narrow environmental niches (Colares et al. 2021). Adults of this species have been commonly collected in montane forests (Silveira et al. 2020), but their larvae have been elusive. Recent fieldwork by our group found photurine larvae dwelling in caves and grottos (small caves) across several spots at the Atlantic Rainforest and the Cerrado biomes. Most of the collections were carried out by RZ as part of various monitoring and research works on cave communities for environmental licensing purposes. The ubiquitous presence of these larvae caught the attention of RZ who managed to raise them to adults and allow us to reliably identify them as *Photuriselliptica*. This is not only the first report of reliably identified firefly larvae in caves, but also the first documentation of these organisms feeding on bat guano. Here, we provide the first description of the larva of *P.elliptica* and document their habitat and feeding behavior. We also provide updated diagnoses and a distribution map for this species.

## ﻿Materials and methods

**Figure 1.**
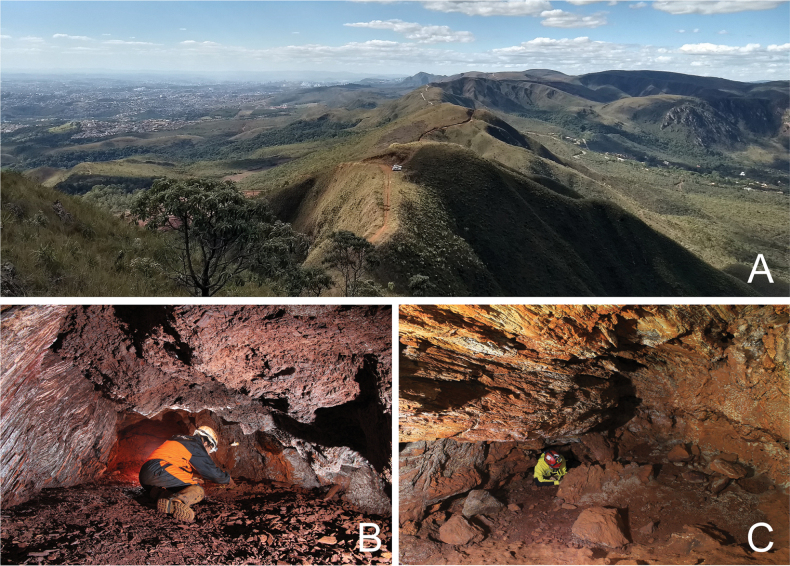
General aspect of the landscape and ferruginous caves in the Quadrilátero Ferrifero, state of Minas Gerais **A** Serra da Moeda **B, C** Caves inserted in the iron formation.

**Figure 2.**
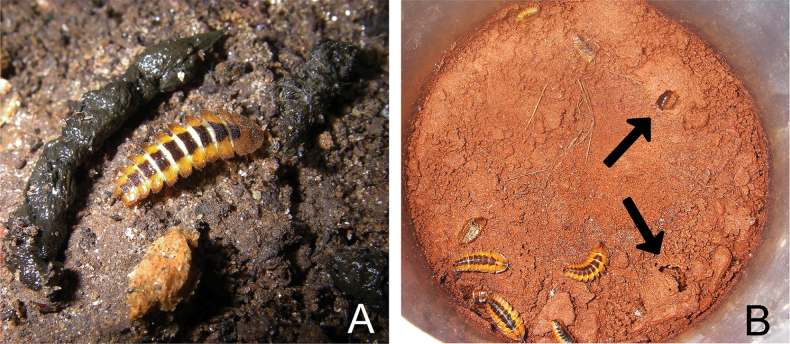
*Photuriselliptica* Olivier, 1886, mature larvae **A** larva eating carnivorous bat guano **B** larvae in the plastic container with fine sediment; arrows indicate pupal chambers.

*Collecting and rearing larvae.* Larvae were collected from the cave RF_0071 (Brazil, Minas Gerais state, Barão dos Cocais municipality) using fine-tip brushes and transported alive to the laboratory in plastic containers with a sample of clay sediments from the cave (Figs 1, 2). The largest last instar larvae were chosen for this procedure to maximize rearing success. The containers were kept at room temperature and light, and the substrate was kept moist. Larvae were fed with fish food until they reached adult forms, which took approximately 30 days. The fish food was predominantly composed of soybean meal, fish meal, creamed corn, and squid meal. The larvae were raised together in the same container without any observed intraspecific predation behaviors (cannibalism). In their last stage, the larvae built chambers in the substrate (Fig. 2B) (as commonly known in other photurine larvae; e.g. Rosa 2007), where they pupated and laid until emerged as adults. On this occasion, we were unable to preserve the pupa before the adult emerged, which is why we did not describe it here.

*Material preparation.* Study of the larval morphology was based on examination of whole specimens and head, mouthparts, and legs dissected after being boiled in water. Dissected larva and whole immature instar specimens were mounted in temporary slides in Hoyer’s medium. Adults were soaked in 10% KOH for 24 hours, then dissected and examined. Drawings were made with a camera lucida adapted to a stereomicroscope Zeiss Discovery V8 or after photographs taken through the eyepiece of a microscope Zeiss Primo Star. Photographs were taken with a Canon EOS Rebel T6 camera with a Canon EF 100 mm f/2.8 lens and Leica M165C, extension tubes, and a LED illumination system (Kawada and Buffington 2016). Images were processed using Helicon Focus 4.03 and Adobe Photoshop 24.2.1 software. The material studied are deposited in the following institutions: Museu de Zoologia da Universidade de São Paulo, São Paulo (**MZUSP**), Coleção de Invertebrados Subterrâneos de Lavras da Universidade Federal de Lavras, Minas Gerais (**ISLA**), Coleção Professor José Alfredo Dutra da Universidade Federal do Rio de Janeiro (DZRJ), and Muséum National d’Histoire Naturelle, Paris (**MNHN**).

*Taxonomy and terminology.* We based our identification on the original description (Olivier 1886) and by comparison to the holotype, deposited at Muséum National d’Histoire Naturelle, Paris, France (**MNHN**). Terminology followed Souto et al. (2019) and Novák (2018) for adults and immature stages, respectively. Accordingly, the terms tergum and epipleura of larva in Rosa (2007) are here corrected for mediotergite and laterotergite, respectively.

## ﻿Results

### ﻿Taxonomy

#### ﻿ Photuris elliptica

Taxon classificationAnimaliaColeopteraLampyridae

E. Olivier, 1886

BBAE3621-472A-54FA-B1BE-B48EC1744863

Figs 2 , 3 , 4 , 5 , 6 , 7 , 8 , 9 , 10 , 11

##### Comparative diagnosis.

**Figure 3.**
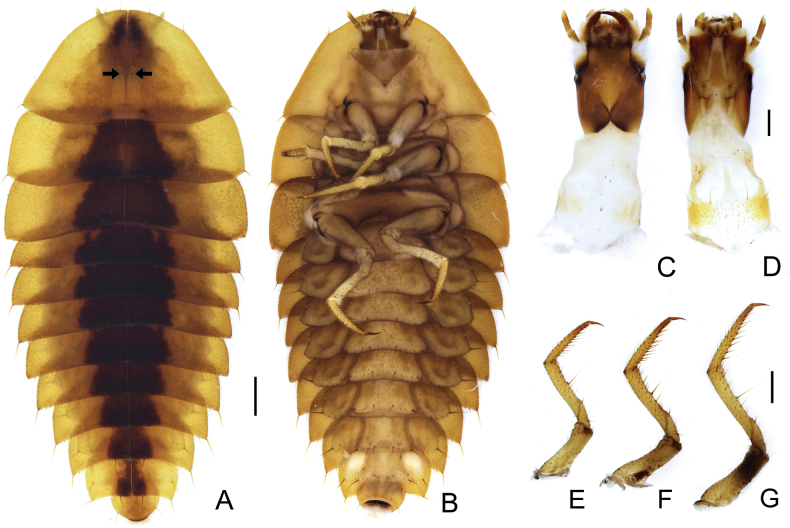
*Photuriselliptica* Olivier, larval morphology, mature larva **A** habitus dorsal view **B** habitus ventral view **C** head dorsal view **D** head ventral view **D–F** right pro-, meso- and metalegs lateral view. Black arrows indicate the parasagittal pair of stout setae. Scale bars: 1.0 mm (**A, B**); 0.5 mm (**C–G**).

**Figure 4.**
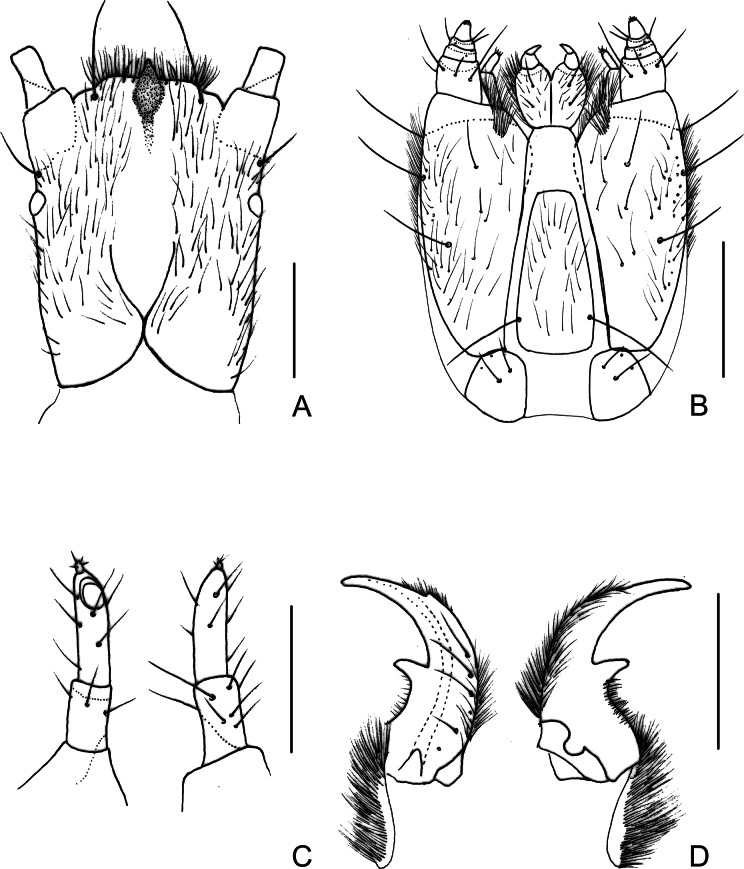
*Photuriselliptica* Olivier, larval morphology, mature larva **A** head dorsal view **B** maxillolabial complex ventral view **C** left antenna ventral and dorsal views **D** right mandible ventral and dorsal views. Scale bars: 1.0 mm (**A, B**); 0.5 mm (**C, D**).

**Larva** (Figs 2–4). Larvae of *Photuriselliptica* are remarkably different from other known congeneric larvae in its color pattern, with thoracic and abdominal mediotergites ochre with black trapezoidal spots medially, which are sometimes medially split (Figs 2A–D, 3A). Other species are brown, reddish brown or black with paler or darker lateral spots (Buschman 1984; Rosa 2007). The chaetotaxy of *Photuriselliptica* and *P.femoralis* is similar, differing by the presence of long, stout setae on anterior margin of pronotum in *P.elliptica* and shape of the longer stouter setae on posterior corners of mediotergite and laterotergite (Fig. 3A, B), which are stiff and acute in *P.elliptica* and subfoliaceous (somewhat flat, tip blunt) in *P.femoralis.* What is more, *P.elliptica* has one pair of parasagittal stouter, longer setae near midlength of pronotum (Fig. 3A) and the ventral stout setae of tibia is longer (about 5 times longer than fine setae) (Fig. 3E), while *P.femoralis* has two parasagittal pairs of setae near midlength of pronotum and ventral stout setae of tibia about 3 times longer than fine setae (see Rosa 2007).

**Figure 5.**
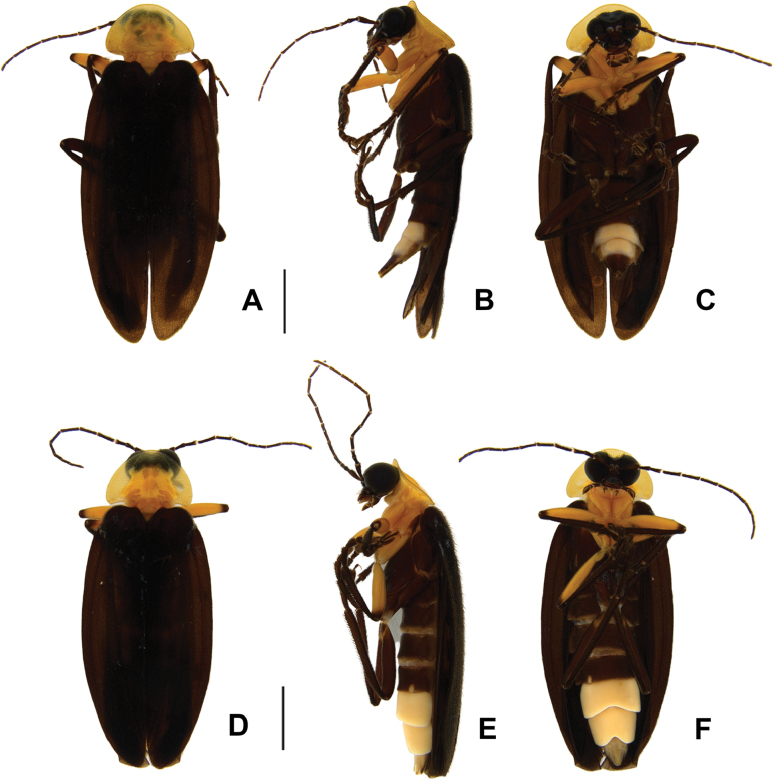
*Photuriselliptica* Olivier, adult habiti **A–C** male habitus: **A** dorsal view **B** lateral view **C** ventral view **D–F** female habitus: **D** dorsal view **E** lateral view **F** ventral view. Scale bars: 2.5 mm.

**Figure 6.**
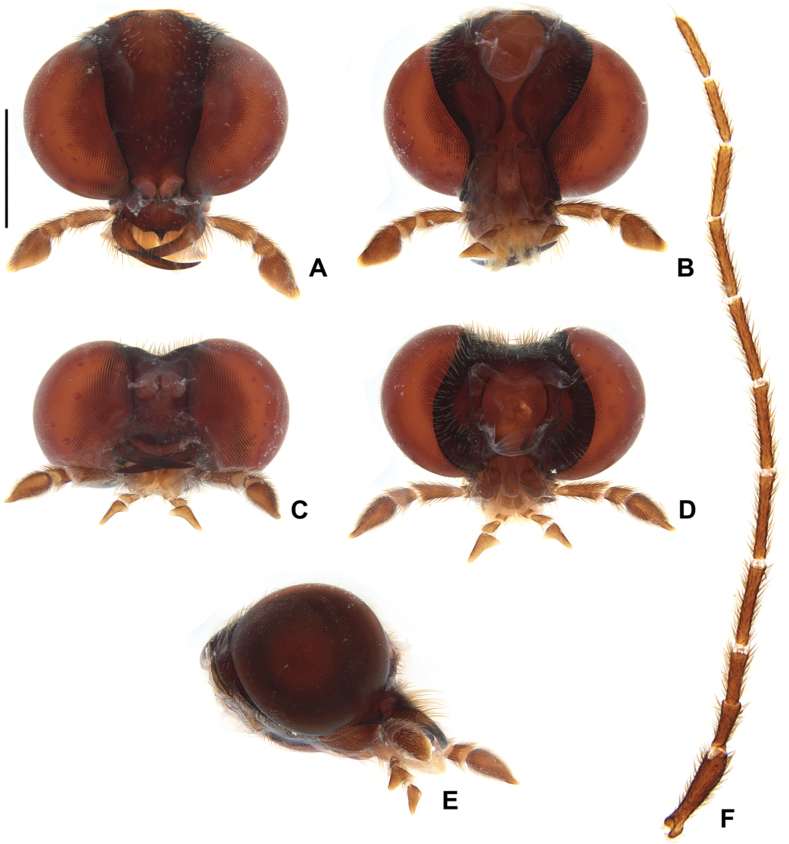
*Photuriselliptica* Olivier, male head **A–E** head capsule: **A** dorsal view **B** ventral view **C** frontal view **D** occipital view **E** lateral view **F** antenna dorsal. Scale bar: 1 mm.

**Figure 7.**
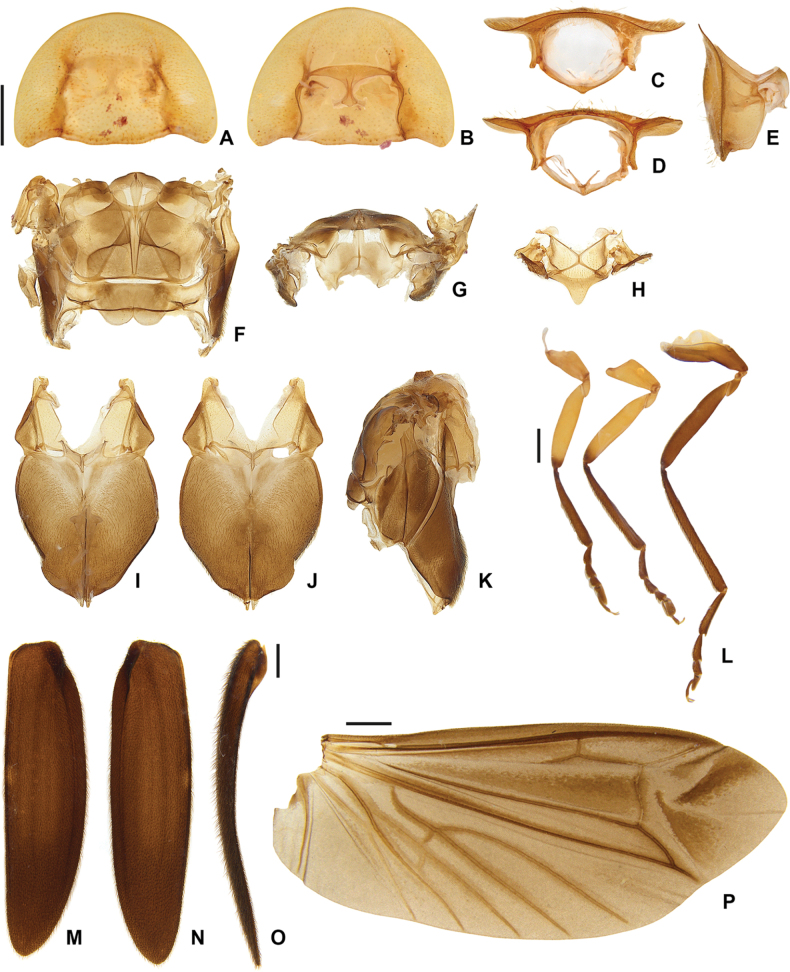
*Photuriselliptica* Olivier, male thorax **A–E** pronotum: **A** dorsal view **B** ventral view **C** anterior view **D** posterior view **E** lateral view **F–G** alinotum: **F** dorsal view **G** anterior view **H** mesoscutellum view **I–K** pterothorax: **I** ventral view **J** dorsal view **K** lateral view **L** proleg, mesoleg, metaleg **M–O** elytron: **M** dorsal view **N** ventral view **O** lateral view **P** wing. Scale bars: 1 mm.

**Figure 8.**
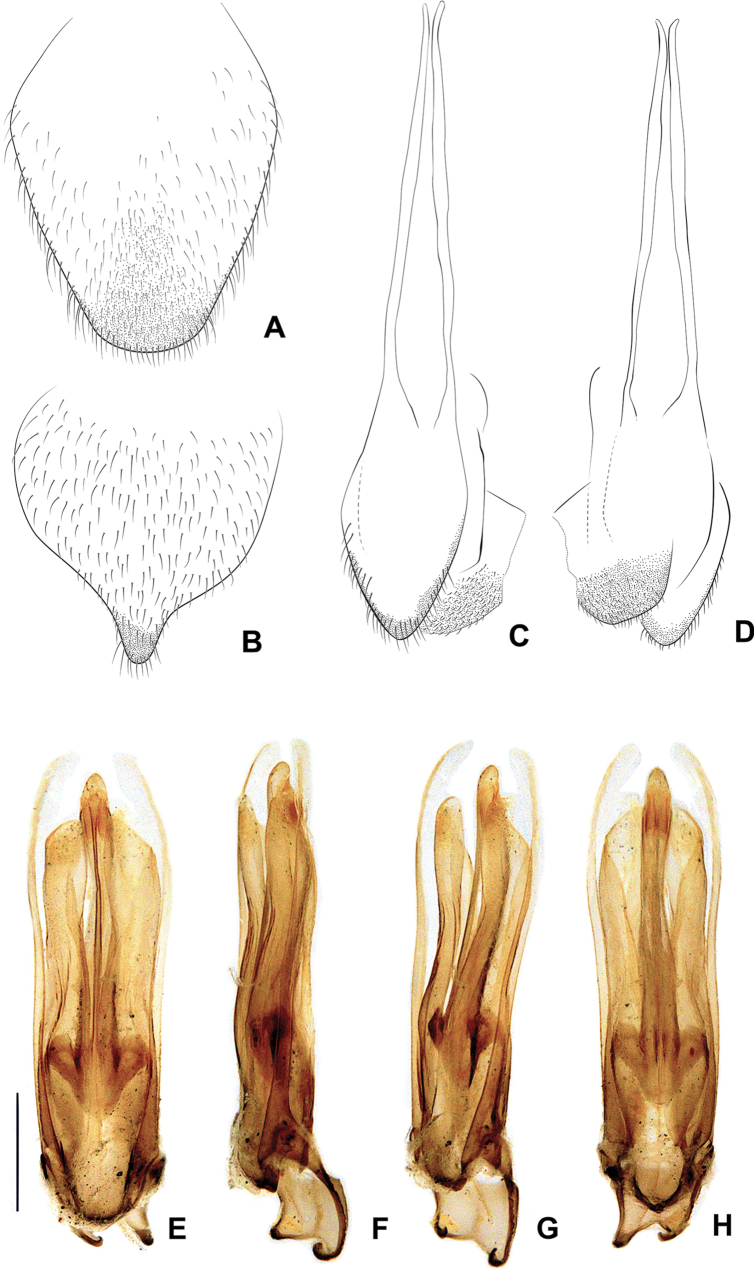
*Photuriselliptica* Olivier, male abdomen **A** pygidium dorsal view **B** sternum VIII ventral view **C** syntergite dorsal view **D** sternum IX ventral view **E–H** aedeagus: **E** dorsal view **F** lateral view **G** oblique view **H** ventral view. Scale bar: 0.5 mm.

**Figure 9.**
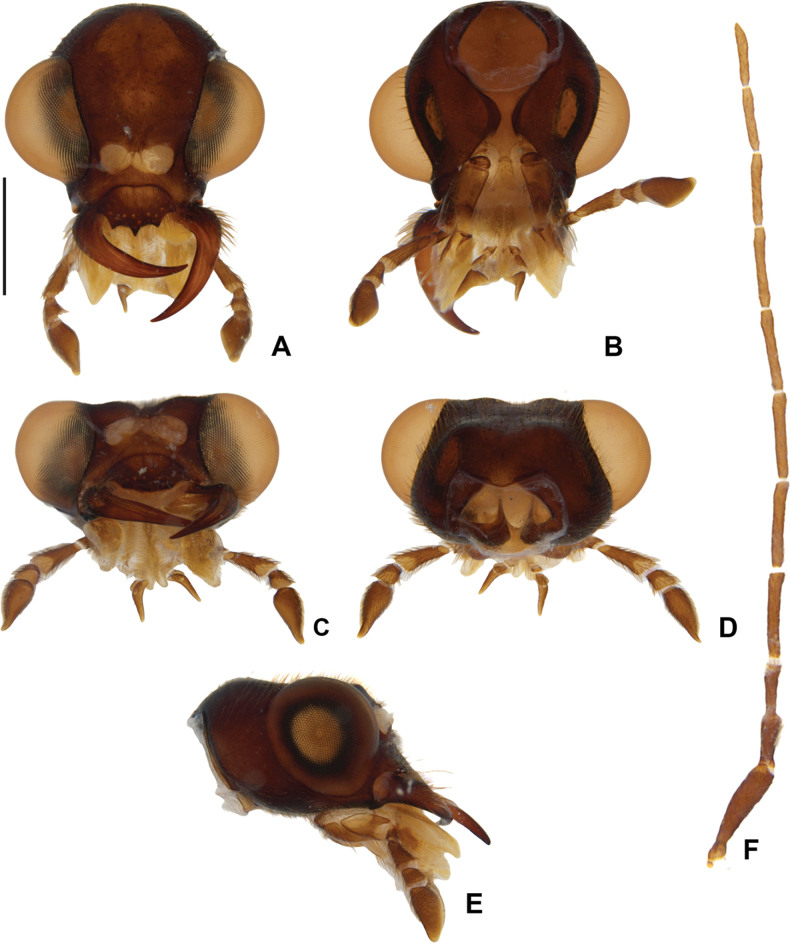
*Photuriselliptica* Olivier, female head **A–E** head capsule: **A** dorsal view **B** ventral view **C** frontal view **D** occipital view **E** lateral view **F** antenna dorsal view. Scale bar: 1 mm.

**Figure 10.**
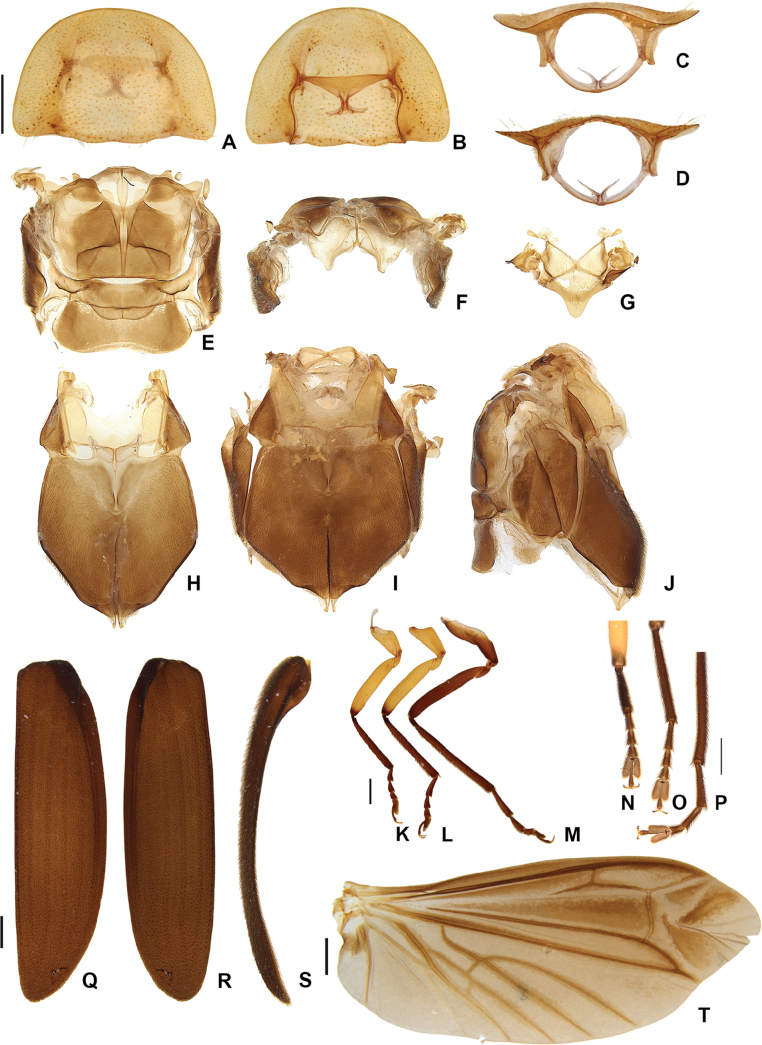
*Photuriselliptica* Olivier, female thorax morphology **A–D** pronotum: **A** dorsal view **B** ventral view **C** anterior view **D** posterior view **E** alinotum dorsal view **F** alinotum anterior view **G** mesoscutellum ventral view **H** meso- and metaventrite ventral view **I** intact pterothorax ventral view **J** intact pterothorax lateral view **K** proleg **L** mesoleg **M** metaleg **N–P** detail of tarsi and claws **N** proleg **O** mesoleg **P** metaleg **Q–S** elytron **Q** dorsal view **R** ventral view **S** lateral view **T** wing. Scale bars: 1 mm.

**Figure 11.**
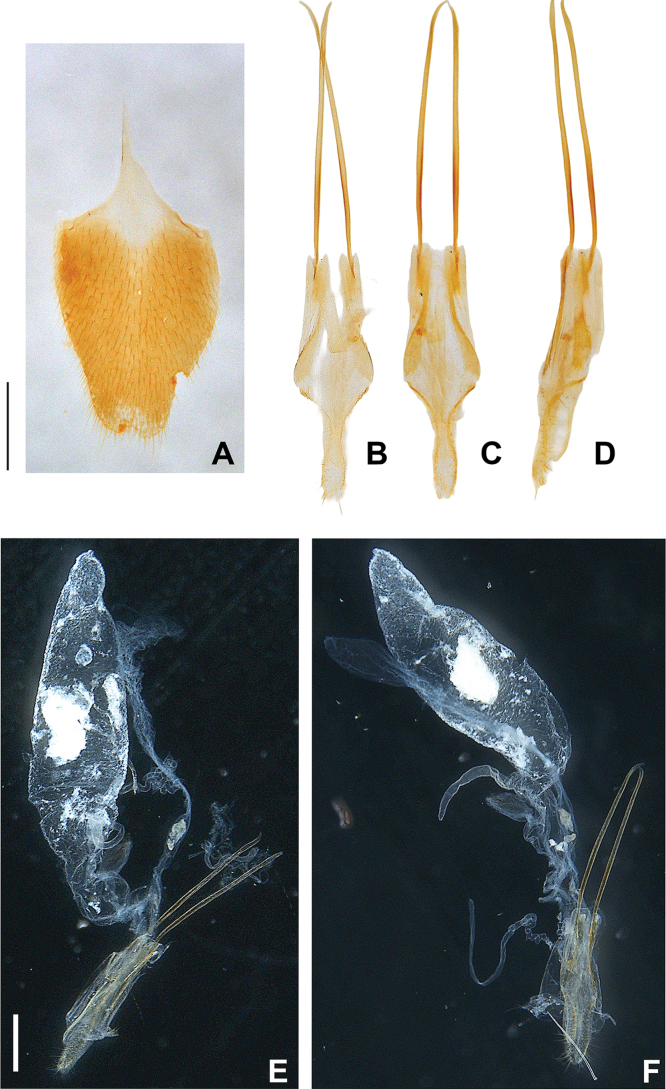
*Photuriselliptica* Olivier, female abdomen **A** sternum VIII ventral view **B–D** ovipositor **B** dorsal view **C** ventral view **D** lateral view **E, F** internal anatomy **E** dorsolateral view **F** lateral view. Scale bars: 0.5 mm.

**Adult** (Figs 5–11). *Photuriselliptica* is very similar to *P.funesta* Gorham, 1880, a common species of the tropical Andes in Colombia (Olivier 1886; LS pers. ob.). Both species share a relatively large size (12–13 mm in *P.elliptica*, ~15–20 mm in *P.funesta*), an overall elongate body and similar color pattern (body dull black, except for the yellow pronotum [with a black spot on the disc in *P.funesta*]. *Photuriselliptica* can be readily distinguished from *P.funesta* by the lack of a black dot at the pronotal disc, obtuse posterior angles of the pronotum (projected and acute in *P.funesta*), and more elliptical elytron (subparallel in *P.funesta*).

In the Atlantic Rainforest of southeastern Brazil, *P.elliptica* is somewhat similar to *P.velox* Olivier, 1886—both species are relatively large, have obtuse posterior corners of the pronotum and elliptical elytra (Silveira et al. 2020). However, *P.velox* has a very different color pattern, with body overall dark brown to black, except for pale yellow pronotal and elytral expansions.

*Photuriselliptica* also overlaps in distribution with *P.femoralis* Curtis and *P.lugubris* Gorham, 1881. *Photuriselliptica* can be distinguished from *P.femoralis* by the elliptical elytral outline (lacking outward lateral expansions in *P.femoralis*) and color pattern (pronotum pale yellow) (Souto et al. 2019; Silveira et al. 2020). *Photuriselliptica* also has a thinner mandible that evenly tapers throughout (larger and constricted by the basal third in *P.femoralis*). *Photuriselliptica* is similar to *P.lugubris*, with a notched posterior margin of the sternal VII, the central tooth on the labrum much longer than the others, and similar color pattern (pronotum yellow, elytron black). *Photuriselliptica* can be distinguished from *P.lugubris* by its yellow pro- and mesocoxae (black in *P.lugubris*), as well as for the more conspicuous marginal costa (less developed in *P.lugubris*).

For overall morphological comparison within the genus, *P.elliptica* shows considerable differences from other *Photuris* with which they do not co-occur, including *P.frontalis* LeConte, 1852, *P.tenusignathus* Zaragoza-Caballero, 1995, and the *P.versicolor* (Fabricius, 1798) complex (Zaragoza-Caballero 1995). Based on the availability of published material and references therein, members of the *P.versicolor* group (including *P.quadrifulgens* (Barber, 1951), *P.trivittata* Lloyd & Ballantyne, 2003, *P.versicolor*, and *P.walldoxeyi* Faust, 2019) are deemed morphologically similar and will be treated as a single group for comparison (Barber 1951; McDermott 1967; Faust and Davis 2019).

*Photuriselliptica* mandibles are thinner and evenly tapered throughout, compared to the thicker mandibles of *P.femoralis* and the *P.versicolor* group which are constricted by the basal third (Fig. 6A). The antennal sockets are very close, nearly contiguous in *P.elliptica* instead of separated by half a socket width in other *Photuris* (Fig. 6C). The labial palp of *P.elliptica* is triangular rather than C-shaped in congenerics (Fig. 6C). The pronotum of *P.elliptica* is wide (1.5 times wider than long) and has a shorter anterior expansion with a distinct dorsal bend as seen in lateral view (Fig. 7A, E), while other *Photuris* feature longer pronota with long, straight anterior expansions. The elytron of *P.elliptica* are also wider, equally wide in the 1^st^ and 2^nd^ thirds, with lateral expansions more pronounced slightly after the humerus (Fig. 7M–O). This is distinct from *P.femoralis*, with straight, narrow elytra, and *P.versicolor* group, with elytra that are slightly convergent throughout. The legs have less prominent trochanters than the other *Photuris* species illustrated in the literature (Fig. 7L). The male lantern covers the entire sterna VI and VII, both of which are much longer than sternum V, unusual for *Photuris* (Fig. 5C). The median projection of the sternum VIII is remarkably longer (a fifth of sternum greater length) than that of *P.femoralis*, *P.lugubris*, and *P.versicolor* group (a sixth), but narrower than *P.frontalis* (roughly a fourth) (Fig. 8B). The posterior margin of the pygidium in *P.elliptica* is truncate (Fig. 8A), similar to *P.versicolor* group, instead of rounded in *P.femoralis*, *P.frontalis*, and *P.lugubris*. The arms of the sternum IX are separated by half the sternum width where it meets the syntergite (Fig. 8C), while the arms in other *Photuris* are separated by a fourth of the sternum IX width or less. Similar to *P.femoralis* and *P.tenusignathus*, and unlike the other ones mentioned, the aedeagus of *P.elliptica* is distinct for lacking the basal lobes at the base of the paramere (Fig. 8H). The tip (apical fifth) of the phallus is also wider (Fig. 8E), similar to *P.frontalis* and *P.lugubris*, rather than constricted in *P.femoralis* and *P.versicolor* group.

Due to lack of published data on *Photuris* females, *P.elliptica* can only be compared to *P.femoralis* (Souto et al. 2019) and *P.versicolor* group (Figs 9–11). The mandibles of *P.elliptica* have smoother inner margins than *P.femoralis* and *P.versicolor* group and are much longer than the latter (Fig. 9A). The median tooth on the labrum is twice as long as the lateral teeth, while *P.femoralis* has teeth all the same size and *P.versicolor* group has a median tooth 1.5 times as long as the lateral teeth (Fig. 9A). The labial palps of *P.elliptica* are less emarginate (less C-shaped) than those of other *Photuris* females (Fig. 9B). *Photuriselliptica* has a slightly depressed vertex of the head (flat in congenerics) (Fig. 9D, E) and antennal sockets that are wider than long (round in congenerics) (Fig. 9C). *Photuriselliptica* and *P.femoralis* also share a wider, shorter pronotum compared to the longer *P.versicolor* group pronotum (Fig. 10A). The *P.elliptica* lanterns are similar to *P.femoralis*, compared with *P.versicolor* group lanterns which are longer and thinner, especially on sternum VI (Fig. 5F). The sternum VIII is lightly sclerotized, similar to *P.versicolor* group, while *P.femoralis* has a strongly sclerotized sternum VIII (Fig. 11A). The arms of the ovipositor in *P.elliptica* are longer than the rest of the ovipositor, resulting in much longer arms than its congenerics (Fig. 11B). Given that no other photurine species have had their bursal anatomy described before, cross-species comparisons are not possible, but we trust even a simple description would help future comparisons. The bursa copulatrix (Fig. 11E, F) of *P.elliptica* has a long and broad spermatophore digesting gland (wider and longer than bursal core), with a basal long and slender pouch, and no distinct bursal sclerites. A spermatheca could not be clearly determined but, if present, it would be very different from other known lampyrid spermathecae (e.g. Fu and Ballantyne 2021; Zeballos et al. 2023).

##### Holotype examined.

Minas Gerais, Caraça, 1/II/1885, male, E. Gounelle col. (MNHN – France, Paris, Muséum National d’Histoire Naturelle). The holotype, confirmed by MNHN curator A. Mantilleri, has the author’s original identification label, but lacks an original type label.

##### Material examined (adults).

Brazil – **Minas Gerais** • 1 ♂; Barão dos Cocais, cave RF_0071; 19°55'21.57"S, 43°30'43.37"W (WGS84); alt. 908 m; 24.III.2014, afótica; Zampaulo R.A. leg.; ISLA without catalog number • 2 ♂; Catas Altas, Vale, Mina Fábrica Nova, cave FN_0001; 20°12'26.69"S, 43°26'18.45"W (WGS84); 18.IX.2020; Eq. Spelayon et al. leg.; ISLA 84748 • 1 ♂; Presidente Olegário, Gruta da Caieira; 18°19'23.99"S, 46°5'16.00"W (WGS-84); 11.X.2010; without collector; ISLA 3102 • 1♂; Arcos/Pains, Agrimg (AGR), 002_001_003; 20°20'21.27"S, 45°34'36.87"W (WGS84); Eq. Spelayon et al. leg.; ISLA 51729 • 1 ♂; Monte Verde; 11.XII.1969; J. Halik leg., MZUSP 9482 • 1 ♀; same localilty; 27.XI.1969; J. Halik leg.; MZUSP 9122 – **São Paulo** state • 2 ♂; Campos do Jordão; 18.XII.1944; F. Lane leg.; MZUSP without catalog number• 1 ♂; Monte Alegre, Fazenda Santa Maria; 1100 m elev.; 28–30.XII.1942; Zoppei & Dente leg.; MZUSP without catalog number• 1 ♂; Santo Antônio do Pinhal (Pindamonhangaba, sic), Estação Eugênio Lefevre; 1200 m elev.; 24.I.1963; Exp. Dep. Zool. leg.; *Photuris*, Silveira det. 2012; MZUSP without catalog number – **Rio de Janeiro** state • 1♂, 1♀; Teresópolis, Parque Nacional da Serra dos Órgãos, represa do Rio Beija-flor; 14–17.I.2015; Silveira leg.; DZRJ 3543.

**Pupa.** Unknown.

##### First instar to mature larva

**(possibly 6^th^ instar) (Figs 2–4).** Body dorsal view (from anterior margin of pronotum to posterior margin of abdominal tergite IX) 2.5–14 mm long, about 2 times longer than wide, oblong (pronotum semicircular, widest at metathorax and gradually decreasing in width posteriorly from abdominal segment III, dorsoventrally flattened. Head dark brown, median region in front of frontal suture paler (Fig. 3C); dorsal surface of body ochre, pronotum medially with a pair of brown elongate spots on anterior half (sometimes almost contiguous), a parasagittal pair of shorter brown spots on posterior margin (sometimes obsolete), mesothoracic, metathoracic and abdominal tergites I–VIII with median trapezoidal brown spot from anterior to posterior margins, about 1/3 as wide as tergite (Fig. 3A, B). Tegument with setae of four types: most surface covered with dense, yellow short decumbent (lying on surface), semi erect and erect setae; edges with few darker stouter and longer setae. (Fig. 3A, B). ***Head*** (Figs 3C, D, 4A, B). About 1.5 times longer than wide (length up to nasale), almost entirely retractable into prothorax (only mandibles and antenna visible in dorsal view when head retracted), sides weakly converging posteriorly, posterior margin with wide triangular notch (Figs 3C, 4A); laterodorsal surface with long, fine setae, one stemma with convex lens laterally at base of antennifer (Fig. 4A); antennifer membranous, as long as basal antennomere (Fig. 4A). Frontal arms V-shaped, well impressed posteriorly, almost reaching ½ length of head (Figs 3C, 4A); epicranial stem very short; clypeolabrum fused to frons, each lateral part darkly sclerotized with anterior edge bisinuous, median part translucent, with dark fusiform plate at middle; plate with anterior part fused to head capsule, forming acute tooth; posterior part fused to epipharynx and visible through translucent cuticle (Fig. 3A, B). Antennae elongate, with three antennomeres, antennomere I partially sclerotized, sparsely setose, cylindrical; antennomere II 1.4–1.7 times longer than I, fully sclerotized, sparsely setose, laterally flattened, apex ventrally with elliptical, flattened sensorium; antennomere III 0.2× as long as antennomere II, attached dorsally to antennomere II, digitiform, subapically one seta and one dome-like projection, apically three spiniform projections (Fig. 4C). Epipharynx with cross-shaped sclerite and two triangular striate plates; plates with anterior margin densely covered with fine setae and two orifices at lateral margins; hypopharynx with anterior part bilobed, densely setose; median part triangular darkly sclerotized, glabrous; posterior part elongate, semitubular. Mandibles symmetrical, falcate, with a channel opening near apex at outer edge, lateroventral edge with dense row of fine setae from base to channel opening; ventromesal margin posteriorly to retinaculum with shorter setae; retinaculum well developed, forming a large, acute tooth; mesal membranous extension densely setose (Fig. 4D). Maxillolabial complex separated from ventral head capsule by narrow membrane; maxillae with cardo as long as wide, 0.25 times as long as stripes; stipites about 2.5 times longer than narrow, with short membranous area on anterior margin, covered with fine setae irregularly distributed, denser laterally, four stouter setae (three laterally, one anteromedially; palpus 4-segmented, tapering toward apex, with sparse fine setae, palpomere I 1.1–1.2 times wider than long, palpomeres II and III transverse, about 1/5–1/4 as long as I, palpomere IV conical 3 times longer than III; galea 2-articulated: basal palpomere triangular, as wide as long; apical palpomere digitiform, 3 times longer than wide, with one stouter long seta apically (as long as palpomere IV) and few shorter setae; lacinia consisting of densely structure connected to dorsomesal stipital edge; labium: prementum covered with fine setae, one stouter setae near each palpus, anterior edge emarginated between palpi, long dark endocarina at midline; palpus two segmented, apical palpomere as long as the basal one, strongly tapered apicad; mentum with anterior 1/3 membranous; posterior 2/3 sclerotized with pair of long setae posteriorly; submentum and gula membranous (Fig. 4B). Post-occipital membrane as long as head, with elongate lateral sclerotization wider and contiguous on prothoracic collar. ***Thorax*** (Fig. 3A, B): dorsal surface covered with short decumbent, semierect and erect setae, tip of posterior angle with one stouter, longer setae (about 4–5 times longer than fine setae), one parasagittal pair of longer stout setae on posterior edge, ventral surface evenly weakly sclerotized, except by darkly sclerotized thin strand at base of coxae (Fig. 3A, B). Pronotum semielliptical 1.6–1.9 times wider than long, posterior margin slightly curved posterad, anterior margin with two pairs anteriorly and one pair anterolaterally of stouter, longer setae (3–5 times longer than fine setae), one pair of parasagittal stouter, longer setae (3 times longer than fine setae) at midlength (Fig. 3A); prothoracic collar weakly sclerotized, ventrally covered with short setae, two pairs of longer, stouter setae anteriorly (Fig. 3C, D); prosternum weakly sclerotized, covered with short setae, each anterior corner with one stouter, longer setae (Fig. 3D). Mesonotum as long as metanotum, both transverse, with anterior and posterior angles almost straight, with transverse pigmented impression parallel to anterior edge (Fig. 3A); mesonotum 3.0–3.3 times wider than long (Fig. 3A); metanotum 3.8–4.0 times wider than long (Fig. 3A); mesepisternum with a functional biforous spiracle on anterior corner. ***Legs*** (Fig. 3E–G): evenly sclerotized, pretarsus darker, with short spiniform setae becoming stiffer and darker from coxa to tarsus, tibia with one longer stouter seta ventrally (about 5 times longer than short setae); pretarsus with one seta on each side at base (Fig. 3D). ***Abdomen*** (Fig. 3A, B) dorsal surface covered with short decumbent, semierect and erect setae, tip of posterior angle with one stouter, longer setae (about 4–5 times longer than fine setae), one pair of longer stouter setae parasagittally; median tergites transverse, gradually narrowed posterad from segment III; I–VIII with anterior angles rounded, posterior angles acute, and transverse pigmented impression parallel to anterior edge, median tergite IX almost circular (dorsal visible part semicircular), about 0.5 times as long as VIII, margin with one pair of stouter longer seta lateroposteriorlly (Fig. 3A). Ventral surface evenly sclerotized, covered with light brown, fine, decumbent and semi-erect setae; laterotergites as long as wide, 0.5–0.8 times as wide as median sternites (widened apicad), inner edge overlapping the lateral edge of the median sternite, posterior edge with 6–7 stouter, longer setae (1.5–2.0 times longer than fine semi erect seta), posterior angle with one stouter, long seta (about 5.0 times longer than fine semi-erect setae), spiracles on lateral edge at midlength; whitish spot (photic organ) occupying almost entirely the laterotergite VIII; median sternites I–VIII trapezoidal, posterior edge with several stouter setae, two pairs of setae 3–4 times longer than fine semi-erect setae (one pair parasagittal, one pair lateral); median sternite IX 1.5 times longer than VIII; segment X ventroapical, membranous, except for a darkly sclerotized transverse strand; pygopodia finger-like, with several dense rows of minutely sclerotized hooks (Fig. 3B).

##### Material examined (larvae).

Brazil – **Minas Gerais** • 19 larvae; Mariana, Vale – Mina Fabrica Nova, cave FN_0005; 20°13'18.36"S, 43°26'2.91"W (WGS84); 2–3.XII.2020; Eq. Spelayon et al. leg; ISLA 83940 (6 larvae 12–14 mm length), ISLA 78905, 83917 [antigo] (1 larvae 4 mm length), ISLA 83939 (5 larvae 10–13 mm length), ISLA 83918 (1 larva 2.5 mm length), ISLA 83913 (1 larva cut in half), ISLA 83919 (1 larva 3.0 mm length), ISLA 83915 (1 larva 11 mm length), ISLA 83916 (3 larvae 4–14 mm); • 6 larvae; same data, but cave FN_0004; 20°13'18.35"S, 43°26'2.63"W (WGS84); 01.XII.2020; ISLA 83934 (1 larva 3 mm length), ISLA 83931 (1 larva 3 mm length), ISLA 83938 (2 larvae 12–13 mm), ISLA 83932 (1 larva 8 mm length) • 1 larva; same data, but cave FN_0027; 20°13'25.55"S, 43°26'15.00"W; 24.IX–09.X.2020; ISLA 83956 (12 mm length) • 1 larvae; same data, but cave FN_0006; 20°13'7.12"S, 43°25'49.72"W (WGS84); ISLA 83957 (10 mm length) • 6 larvae; same data, but cave FN_0025; 20°13'0.57"S, 43°26'35.61"W (WGS84); 24.IX–30.X.2020; ISLA 83947 (1 larva 13 mm length), ISLA 83945 (2 larvae 11–12 mm length), ISLA 83903 (1 larva 11 mm length), ISLA 83946 (2 larvae 10–13 mm length) • 6 larvae; same data, but cave FN_0003; 20°13'19.20"S, 43°26'2.76"W (WGS84); 03–04.XII.2020; ISLA 83926 (1 larva 2.5 mm length); ISLA 83924 (5 larvae10–13 mm length) • 2 larvae; same data, but cave FN_0002; 20°13'38.49"S, 43°25'52.23"W (WGS84); 04.XII.2020; ISLA 83937 (1 larva 4 mm length), ISLA 83935 (1 larva 11 mm length) • 7 larvae; Dores de Guanhães, G. Energia, cave CAV 05; 19°1'32.90"S, 42°53'27.24"W (WGS84); 30.I–03.II.2017; Eq. Spelayon et al. leg.; ISLA 52343 (11–13 mm length); • 1 larva; same data, but 29–31.V.2017; ISLA 52341 (13 mm length); • 2 larvae; same data but CAV 008; Lat. 19,0640/Long. 42,9270; ISLA 52344 (7–9 mm length) • 1 larva; same data, but cave DGN005; 19°2'25.09"S, 42°51'54.36"W (WGS84); 11–15.XII.2015; ISLA 45434 (7–10 mm length) • 1 larva; same data but, Energia cave SPT 004; 19°1'42.61"S; 42°55'27.13"W (WGS84); 11–12.XII.2015; ISLA 45513 (8–10 mm length) • 1 larva; same data, but G.E.-S2_NOVA 004; 18°58'59.54"S, 42°55'40.10"W (WGS84); 5–7.VII.2016; ISLA 45596 (5 mm length); • 3 larvae; same data but NOVA_003; 18°59'28.68"S, 42°55'57.75"W (WGS84); ISLA 45595 (6–12 mm length); • 1 larva; same data, but G. Energia SPT 004; 19°1'42.61"S, 42°55'27.13"W (WGS84); 17–20.VII.2015; ISLA 45514 (9 mm length); • 1 larva; same data, but G.Energia, cave DGN 005, DGN005; 19°2'25.09"S, 42°51'54.36"W (WGS84); 19–21.VII.2015; ISLA 45436 (10 mm length) • 2 larvae; Barão dos Cocais, cave CAV 01; 19°59'53.63"S, 43°33'56.52"W (WGS84); 06.III.2016; Fábio Bondezan leg; ISLA 47284 (13 mm length); • 1 larva; same data, but cave RF_0092; 19°55'51.44"S, 43°31'47.09"W (WGS84); 18.IX.2014; Eq. Ativo Ambiental leg.; ISLA 471 (12 mm length); • 1 larva; same data, but CAV 11; 20°0'21.44"S, 43°34'4.08"W (WGS84); 7.III.2016; ISLA 47283 (5 mm length); • 1 larva; São Gonçalo do Rio Abaixo, VALE Brucutu, cave BRU_0002; 19°53'21.55"S, 43°26'16.11"W (WGS84); 16.V.2020; Spelayon et al. leg.; ISLA 81940 (7 mm length); • 1 larva; same data, but cave BRU_0008; S 19°52'33.74"S, 43°25'3.11"W (WGS84); 19–23.VIII.2020; ISLA 82162 (11 mm length); • 1 larva; Santana do Riacho, Gruta da Viola; 19°17'44.67"S, 43°37'0.33"W (WGS84); 17.IV.2017; Proj. MG/Rabelo et al. leg.; ISLA 78921 (12 mm length); • 5 larvae; Coração de Jesus, Gruta Sumitumba; 16°39'47.90"S, 44°22'8.42"W (WGS84); 29.I.2015; ISLA 78709 (3 larvae 3–5 mm, 2 larvae 13 mm length) • 1 larva; Lima Duarte, Parque Estadual do Ibitipoca, Gruta Manequinho; 21°43'11.64"S, 43°54'11.16"W (WGS84); ISLA 78708 (10 mm length); • 1 larva; Rio Pardo de Minas, Peixe Bravo, cave Lago; 15°59'55.17"S, 42°44'42.63"W (WGS84); ISLA 78904 (13 mm length).

##### Biology and life cycle.

The larvae of *Photuriselliptica* were collected only inside caves located in different lithologies, mainly ferruginous rocks (mostly), but also limestone, quartzite, and granite (see above). In general, the larvae are found in aphotic zones, under blocks or on the surface where the floor is formed by fine sediment (sand or clay), places where it is possible to build chambers for their metamorphosis. Regarding food, larvae were observed feeding on guano from insectivorous, carnivorous, and hematophagous bats (Fig. 2). Although immature forms are recurrent in caves, adult forms are rarer, and adults are therefore expected to disperse to surface environments after hatching. Inside the cave, bioluminescence was quite difficult to observe. The larvae emitted a very faint greenish light for only a few seconds and then went for a long time without emitting light. The light from the flashlights and human approach (disturbance) seemed to inhibit the larvae from glowing. There were a few observations of luminescence, just after remaining still and keeping the flashlight off for several minutes.

Many larvae of different sizes were collected, but only mature larvae (those one 12–14 mm length) were reared until adult stages, and, thus, we could not count the exact number of instars. Still, compared with *Photurisfemoralis*, *P.elliptica* is a little smaller (*P.femoralis* first instar larva is 2.7 mm, 6^th^ instar larva 12.2 mm, adults 10.0–10.6 mm, while *P.ellyptica* larvae ranged from 2.5–14.0 mm and adults 12.0–13.0 mm length), suggesting that *P.elliptica* has the same number of larval instars as *P.femoralis* (usually six, rarely seven instars). Thus, we probably examined all larval instars, being first instar 2.5–3.0 mm length and sixth 13.0–14.0 mm length. What is more, this indicates that at least the entire larval stage occurs inside caves.

##### Distribution

**Figure 12.**
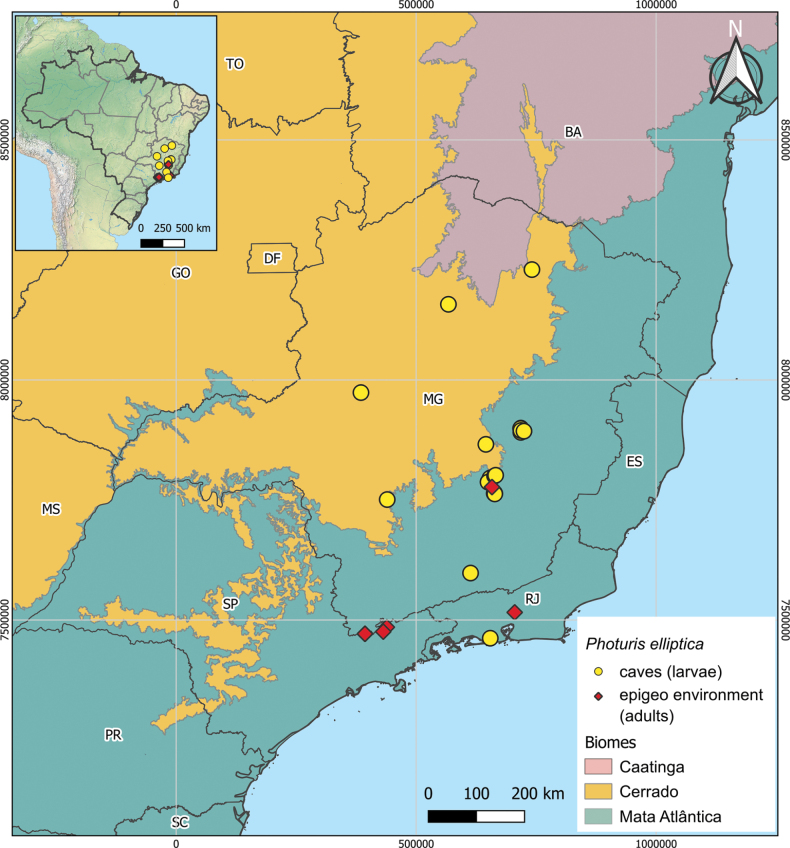
Political and biogeographic map of Brazil, showing the spatial distribution of *Photuriselliptica* Olivier, which occurs in two different Brazilian continental biomes, the Mata Atlântica and Cerrado. Letters on the map correspond to Brazilian states. Abbreviations: BA, Bahia; DF, Distrito Federal; ES, Espírito Santo; GO, Goiás; MG, Minas Gerais; MS, Mato Grosso do Sul; PR, Paraná; RJ, Rio de Janeiro; SC, Santa Catarina; SP São Paulo

**(Fig. 12).** Most of the observations of the species were made in caves (larvae) and surface ecosystems (epigean) located in mountainous areas at altitudes of above 1000 m. However, some occurrences were observed in regions at lower altitudes in the north and center-west regions of Minas Gerais. Furthermore, *P.elliptica* species can be found in the Atlantic Forest and Cerrado biomes, located in the states of São Paulo, Rio de Janeiro, and Minas Gerais, Brazil.

## ﻿Discussion

### ﻿Are *Photuriselliptica* larvae cave specialists?

Caves have unique environmental conditions that set them apart from surface ecosystems. These conditions include higher humidity, the complete absence of light, and a lower availability of food (Poulson and White 1969; Culver 1982). Thus, cave ecosystems are selective environments where only species with morphological, physiological, or behavioral pre-adaptations can successfully colonize and establish viable populations over time (Culver 1982). However, caves are attractive environments due to the scarcity of specialized predators (Gibert and Deharveng 2002; Fernandes et al. 2016) and, thus, are ideal for laying and development of eggs of those species able to survive in these environments.

Lampyrid larvae occupy a wide array of environments (see above; reviewed by Riley et al. 2021), but our observations are to our knowledge the first report of a lampyrid larvae dwelling in caves. A few traits of this species’ larva may be adaptations to a cave life. For instance, the brighter, less pigmented *P.elliptica* larval color pattern could be the outcome of relaxed selection for camouflage patterns in the aphotic zone of caves (Fig. 2). Similar phenotypes are common in cave beetles (e.g. Luo et al. 2018). Likewise, the longer leg setae (Fig. 3E–G) could indicate greater reliance on chemical and physical cues, compared to dwellers of open environments, as found elsewhere in beetles (e.g. Luo et al. 2023). Both observations are yet to be tested by field observations and experiments. Yet, the broader diet of *Photuris* larvae may be a key factor allowing their widespread occurrence in caves.

Caves are oligotrophic environments, with limited availability of food items, partly due to lack of light and, consequently, of photosynthetic organisms (Culver and Pipan 2009). Therefore, cave food webs depend on their connectivity to surrounding surface environments (Kováč 2018). In this context, bat guano is a key source of energy for cave environments.

*Photuris* are unique among lampyrid larvae in having a comparatively broader menu. Most firefly larvae specialize in gastropods and or/earthworms, whereas *Photuris* larvae will readily eat arthropods, and even plants. For example, Buschman (1984) reported 21 food records from field observations for *Photuris* larvae: five were snails and slugs, 11 were insects (caterpillars, membracids, adult cerambycids, and dipteran larvae), four were fallen berries, and one was an earthworm. Likewise, Faust and Faust (2014) reported *Photuris* larvae eating milkweed rhizomes —a chemically defended plant—and no adverse reactions were observed. All *P.elliptica* larvae in the field were seen eating bat guano, of different kinds (see above), and nothing else, despite the presence of slugs and earthworms. However, it cannot be ruled out that these larvae have a broader menu. In fact, it is yet unknown whether guano is even a preferred rather than tolerated food item. Nevertheless, the fact that these larvae can live on bat guano for several weeks, until they managed to successfully pupate and emerge from the pupa, may facilitate their occurrence in caves.

Most of the larvae analyzed in the present work were collected in ferruginous caves. A possible gateway to caves for *Photuris* larvae would be the roots of trees or even the natural porosity of the rock, especially in iron ore caves, which are often relatively shallow or close to the surface (Ferreira et al. 2015). Thus, generalist organisms such as *Photuris* larvae could easily access and colonize underground environments.

We therefore encourage future firefly surveys to include underground environments, hoping that this will help mitigate the staggering knowledge shortfall on lampyrid larvae, as well as provide a better understanding of the ecological and evolutionary condition of the use of these environments by firefly species.

## ﻿Conclusions

*Photuriselliptica* larvae dwell in caves of differing lithologies, where they were observed to feed on bat guano of diverse compositions. Although these larvae have some interesting deviations from other known *Photuris* larvae—including lesser pigmentation and unique or longer setae—it is yet unclear whether they are cave specialists. *Photuriselliptica* adults were rarely seen and are yet to be collected in caves, although they are locally abundant elsewhere in forested sites of the Atlantic rainforest.

## Supplementary Material

XML Treatment for Photuris elliptica

## ﻿Additional information

### Author ORCIDs

Paula M. Souto https://orcid.org/0000-0001-6995-9146

Luiz F. L. da Silveira https://orcid.org/0000-0002-0648-3993

### Data availability

All of the data that support the findings of this study are available in the main text.
